# Reply to Comments on “Efficient full-path optical calculation of scalar and vector diffraction using the Bluestein method”

**DOI:** 10.1038/s41377-020-00448-8

**Published:** 2021-01-11

**Authors:** Yanlei Hu, Zhongyu Wang, Xuewen Wang, Shengyun Ji, Chenchu Zhang, Jiawen Li, Wulin Zhu, Dong Wu, Jiaru Chu

**Affiliations:** 1 CAS Key Laboratory of Mechanical Behavior and Design of Materials, Key Laboratory of Precision Scientific Instrumentation of Anhui Higher Education Institutes, Department of Precision Machinery and Precision Instrumentation, University of Science and Technology of China, 230026 Hefei, China; 2 Department of Mechanical Engineering and Department of Civil and Environmental Engineering, Massachusetts Institute of Technology, Cambridge, MA 02139 USA; 3 State Key Laboratory of Advanced Technology for Materials Synthesis and Processing, International School of Materials Science and Engineering, Wuhan University of Technology, 430070 Wuhan, China; 4 Institute of Industry and Equipment Technology, Hefei University of Technology, 230009 Hefei, China

**Keywords:** Optics and photonics, Optical physics

Dear Editor,

In ref. ^1^, we present an efficient full-path optical calculation by using the Bluestein method. A real optical apparatus for laser processing, imaging, or optical tweezing normally involves diverse optical lenses with different physical and numerical apertures (NA). In such applications, the optical path is usually tortuous and long. To assist in the design, evaluation, and alignment of optical instruments, it is advantageous to retrieve the optical field in an arbitrary position along the entire optical path, which is termed the full-path calculation in our paper, with sufficient accuracy and efficiency. In particular, high flexibility is required to accommodate the mismatch between optical apertures of different components in the optical path. We present a full-path optical calculation method by adopting the Bluestein method to address this realistic demand.

The Bluestein method was first developed by Bluestein when working with chirp filtering by converting a discrete Fourier transform (DFT) for non-power-of-two signals into an efficient convolution^2,3^. Soon afterward, this approach was further extended by Rabiner et al. into the chirp-z transform (CZT) algorithm as a generalization of DFT^4,5^. Since then, the Bluestein method (CZT algorithm) has emerged as an important mathematical tool for fast Fourier transforms (FFTs). The Bluestein-based CZT algorithm has been employed in different scenarios of optical calculations since it was established. For example, in 1984, the chirp-z transform was first implemented by Glaser et al.^6^ in an incoherent optical two-dimensional Fourier transform. In 2002, Bakx^7^ adopted the CZT algorithm for the computation of DVD disk readout signals. Restrepo and Garcia-Sucerquia utilized the unprocessed Bluestein transform for magnified reconstruction of digitally recorded holograms^8^. All of these works were developed by using Bluestein’s FFT method to solve specific issues in optical calculations. We are sorry that these related works have not been noticed by us until recently. We thank the enthusiastic readers for bringing these to our attention.

It is claimed in the commentary that Eq. 12 in ref. ^1^ resembles Eq. 18 in ref. ^9^. Actually, the formula in both papers originates from Rabiner’s original paper (Eqs. 4 and 7 in ref. ^4^, which is cited as ref. 23). The same formula can also be seen in ref. ^7^ as Eq. 12. As Bluestein and Rabiner et al. laid the mathematical foundation for the efficient computation of the Fourier transform, the method can be used as long as the calculation can be formulated by the Fourier transform.

We are grateful to Dr. Y. Shao and Prof. H. P. Urbach for pointing out that ref. 15^9^ utilized the CZT algorithm to compute the FFT for focus field calculations. We sincerely apologize for the inaccurate description of the references, and this issue will be described in the last paragraph as an author correction. We are also sorry for the lack of the introduction on the application of the Bluestein method in optical calculations. The CZT algorithm is introduced in section 3.5 in ref. ^9^ for the generalization of the FFT. We greatly appreciate the elegant work by Leutenegger et al. However, the frame of our paper for full-path optical calculations is beyond the scope of ref. ^9^. The paper by Leutenegger et al. considered only the vector diffraction scenario focused by a high NA lens. Full-path light propagation through numerous lenses and elements in complex optical systems are not covered.

In our work^1^, the full-path calculation method for light propagation by interconnecting scalar and vector diffraction is presented. Scalar diffraction and vector diffraction are both computed by the Bluestein method in two dimensions. The efficiency and flexibility of the Bluestein method are fully exploited to realize dynamic aperture matching during light propagation, thus making efficient full-path calculations feasible for realistic implementation. The performance of this method is systematically investigated, and its capacity in evaluating realistic optical instruments is demonstrated numerically and experimentally. Therefore, the full-path calculation is the main contribution of this work as discussed in the last two paragraphs in the introduction section.

For some other issues, a series of modifications and optimizations are made to the Bluestein method to retrieve correct phase patterns and to achieve maximized computational efficiency. The codes can be found in the GitHub repositories, as indicated in the supplementary information. To investigate the accuracy and efficiency conveniently, the most representative examples, such as the point spread function of converging spherical light and focused optical vortex, which have been the “Drosophila” in optical calculations, are chosen in the paper to facilitate a comparison with the results obtained by other methods (e.g., refs. 13 and 17 in the original paper). Unconventional three-dimensional FT (3D-FT) can be derived (refs. 16 and 17) and is also able to be implemented by CZT to directly calculate the volumetric optical field. In comparison, fast two-dimensional (2D) computation is applied in our method because planar optical field information is required for efficient full-path light calculations. In scalar diffraction, although the polarization of the light is ignored and the paraxial condition is assumed during propagation, the nonparaxial light field with complex polarization can be handled by dividing the light into two orthogonal polarization components that propagate independently and then analyzed using the Rayleigh–Sommerfeld diffraction integral, which can also be computed using the Bluestein’s FFT method. In vector diffraction, the calculation of the electromagnetic components (intensity, phase, and polarization) along three orthogonal directions is conveniently obtained by introducing the polarization transform matrix.

Since the application of Bluestein’s method in the optical calculation is inaccurately described in the introduction section, the authors have made the following corrections. On page 2, “Fast Fourier transform (FFT)-based algorithms have been developed to perform fast calculations of light diffraction^15–19^. However, these methods can generate only the light field distribution within a fixed region of interest (ROI) and sampling numbers (i.e., resolution) determined by the intrinsic characteristic of the Fourier transform (FT), lacking flexibility in computing the desired local distribution with variable sampling intervals” should be changed to “Fourier transform (FT) and Bluestein-based algorithms have been developed to perform fast calculations of light diffraction^15–19^”. Among them, the original fast Fourier transform (FFT) methods can generate only the light field distribution within a fixed region of interest (ROI) and sampling numbers (i.e., resolution) determined by the intrinsic characteristic of the method; hence, these approaches lack flexibility in computing the desired local distribution with variable sampling intervals. Bluestein’s FFT method is emerging as a promising approach for optical computations of the focus field, whose efficiency and flexibility can be fully exploited for universal implementations to make the statement more accurate. In addition, on page 3, ref. 15 should be cited again in front of Eq. 10.

We apologize for any inconvenience this may have caused.
